# Forecasting the Concentration of Particulate Matter in the Seoul Metropolitan Area Using a Gaussian Process Model

**DOI:** 10.3390/s20143845

**Published:** 2020-07-09

**Authors:** JoonHo Jang, Seungjae Shin, Hyunjin Lee, Il-Chul Moon

**Affiliations:** Department of Industrial and Systems Engineering, Korea Advanced Institute of Science and Technology (KAIST), Daejeon 34141, Korea; adkto8093@kaist.ac.kr (J.J.); tmdwo0910@kaist.ac.kr (S.S.); uhyh9002@kaist.ac.kr (H.L.)

**Keywords:** particulate matter, forecasting model, dispersion model, PM2.5, PM10, Gaussian process, ARIMA

## Abstract

Recently, the population of Seoul has been affected by particulate matter in the atmosphere. This problem can be addressed by developing an elaborate forecasting model to estimate the concentration of fine dust in the metropolitan area. We present a forecasting model of the fine dust concentration with an extended range of input variables, compared to existing models. The model takes inputs from holistic perspectives such as topographical features on the surface, chemical sources of the fine dusts, traffic and the human activities in sub-areas, and meteorological data such as wind, temperature, and humidity, of fine dust. Our model was evaluated by the index-of-agreement (IOA) and the root mean-squared error (RMSE) in predicting PM2.5 and PM10 over three subsequent days. Our model variations consist of linear regressions, ARIMA, and Gaussian process regressions (GPR). The GPR showed the best performance in terms of IOA that is over 0.6 in the three-day predictions.

## 1. Introduction

Recently, the population of Seoul was affected by fine dust or particulate matter (PM) in the atmosphere [1]. Although some conjectured that the PM originated from outside metropolitan area [2,3,4], others also emphasized on sources such as traffic, the human activity, and the chemical reactions in the atmosphere in the area [5,6]. In addition to the problem of the sources, the dynamics of PM needs to be modeled to aid the prediction of the concentration of PM to address the exposure to the population.

As we cannot determine the main source of PM, the model needs to consider PM generation from a holistic perspective and the factors of the dynamics of the PM concentration. These perspectives and factors are not limited to a single domain of expertise such as traffic, chemistry, meteorology, and environmental studies. Therefore, we enumerate potential factors involved in PM concentration prediction. We present the relative significances of the factors with regard to Seoul.

Using these varieties of inputs, we model the concentration with two different statistical models: Autoregressive integrated moving average (ARIMA) and Gaussian process (GP). These models are applicable to regression tasks in continuous domains with continuous outputs. The types of inputs and the outputs are consistent with our application. Particularly, we employ the Gaussian process because of its nonlinearity of the output. Our analyses of the inferred model consist of two folds. First, we evaluate the prediction performance of the model with the index-of-agreement (IoA) and root mean squared error (RMSE). Second, we examine the relative strength and the interpretation of the coefficients from the inferred models to identify the most significant factors in determining the PM concentration.

## 2. Previous Research

### 2.1. Development on Prediction Model Structure

This section discusses Table 1 by classifying the models by their complexities. Although linear regression and its variants were frequently used, neural networks have recently been explored. This paper presents a prediction model with a Gaussian process regression that is a nonlinear and nonparametric regression model.

#### 2.1.1. Prediction with a Linear Model

This subsection reviews the linear regression models and its variants in Table 1 with focus on the PM concentration prediction. Because PM concentrations are significantly dependent on meteorological conditions, most studies have proposed models to investigate the relation between PM concentrations and meteorological data. Garcia et al. [8] proposed a generalized linear model (GLM) to predict PM10 concentrations in an urban area, whose size is approximately that of a medium-scale city with an area of 34 km^2^. The proposed model utilized (1) air quality data such as CO, NO_2_, NO, O_3_, SO_2_, and PM10; and (2) meteorological data, such as temperature, relative humidity, and wind speed. The coefficients of the linear model were analyzed to determine the effects of each input dimension with respect to the prediction of PM10.

#### 2.1.2. Prediction with a Neural Network Model

This subsection describes the neural network-based approaches in Table 1 for PM concentration prediction. Recently, researchers have adopted neural network models to approximate the nonlinearity of the concentrations. Zhou et al. [12] utilized air pollutant CO, NO_2_, O_3_, SO_2_, PM2.5, and PM10 and meteorological data (temperature, relative humidity, wind speed, and wind direction). Because the concentration of PM2.5 exhibits complex nonlinear dynamics, the study proposed a recurrent fuzzy neural network (NN) for the prediction. To select important factors from several factors, the model utilized the partial least square (PLS) algorithm. Therefore, the proposed neural network comprised a membership function, rule, defuzzy, and output layers. The proposed model demonstrated accurate predictions because the model uses the dynamic information from past records. Zhao et al. [15] proposed a long short-term memory-fully connected (LSTM-FC) neural network for predicting the PM2.5 concentration in a metropolis. The inputs of this model consist of air quality data, meteorological data, and the day of the week. This predictive model is developed on two components. One component is a model on the local variation of PM2.5 from an LSTM-based temporal simulator. The other component considers spatial dependencies among stations using a neural network-based spatial combinator. The combination of these components revealed that LSTM-FC outperforms the vanilla versions of NN and LSTM because it can memorize a long-term dependency. To consider the spatio-temporal dependency, Pak et al. [17] proposed a neural network model, called CNN-LSTM, with two components. The first component is a spatio-temporal convolutional neural network (CNN), and the second component is an LSTM model. The CNN-LSTM predicts the daily average PM2.5 of the subsequent day. CNN-LSTM showed that LSTM outperforms a simple MLP because LSTM is efficient in considering the long-term information of the input data. The CNN-LSTM also showed that the prediction performance improves if CNN is combined with LSTM because CNN extracts the inherent features of the input data.

#### 2.1.3. Prediction with a Nonlinear and Nonparametric Regression Model

This subsection describes the existing studies on the Gaussian process regression-based models in Table 1. Our approach is in this category of PM concentration prediction model. Several studies have used Gaussian Process Regression (GPR) to predict PM concentrations [18,19,20]. Cheng et al. [18] proposed a GPR model to predict the PM2.5 concentrations at locations where the concentration was not observed, utilizing the concentration data from the monitoring sites. Whereas most studies focused on investigating the relation between PM concentrations and input features, such as meteorological data, this study focused on estimating PM2.5 concentration that are not observable owing to the lack of the monitoring sites. Reggente et al. [19] proposed a GPR model to predict ultrafine particle (UFP) concentrations, also called PM0.1 concentrations. The proposed GPR model utilized the air quality data (CO, NO_2_, NO, O_3_) from three monitoring sites in an urban area, approximately a small-scaled city with size 3.93 km^2^. This study empirically demonstrated that GPR outperforms Bayesian linear models. Furthermore, they showed that GPR that uses NO and NO_2_ as covariates, outperforming models that use CO and O_3_ as covariates. Liu et al. [20] proposed a GPR model that combines squared-exponential and periodic kernels to predict PM2.5 concentrations of the subway indoor air quality. This study utilized air quality (CO_2_, CO, NO_2_, NO) and meteorological data, such as temperature and humidity. From the experiments on varying cases of kernel combinations, they empirically demonstrated that optimal performances are obtained from the combination squared-exponential and the periodic kernels. Our study involves developing a GPR model that uses comprehensive input features, such as topography, traffic, and coal-based power generation, and a kernel function that combines the Matérn and the periodic functions that have not been tested in existing studies.

### 2.2. Integration of Societal and Urban Information into Prediction

In addition to the meteorological data, researchers have utilized data produced by residents and the geographical features of the city. Lu et al. [11] utilized the traffic data to focus on the PM concentration at urban intersections. The calculation of the traffic volume is based on each green-light period, and the PM concentrations are collected for the corresponding green-light period. This study proposed a novel hybrid model combining an artificial neural network (ANN) model and a chaotic particle swarm optimization (CPSO) algorithm. The CPSO algorithm is used to overcome the overfitting problem of ANN and to prevent local minima. Based on the relation among the background PM concentration, traffic data, and meteorological data, the combination of ANN and CPSO outperforms the ANN model. Additionally, the study demonstrated that wind speed in winter plays an important role in the prediction of PM at urban intersections. Lal et al. [10] focused on pollution from open-casting mines because air pollution has a significant impact on the health of mining workers and those living near mines. An ANN-based model was developed to predict the PM10 and the PM2.5 concentrations using the meteorological data (wind velocity, dispersion coefficients, rainfall, cloud cover, and temperature), the geographical data, and the emission rate as inputs. Whereas most studies focused on the effect of meteorological and air pollutant data on PM, Zhang et al. [9] utilized land-use data as an input. The inputs contain traffic and population data. The land-use data consist of farmland, forest, grassland, water, urban, and rural areas. The traffic data include the distribution of road networks. Using these inputs, Zhang et al. proposed a spatio-temporal land-use regression model, and investigated the correlation between PM2.5 and the inputs including land-use.

To strengthen the geographical features, researchers have used remote sensing information in the prediction. Observations from ground-level monitoring sites have limited spatial coverage. Therefore, the limited observation does not accurately indicate the spatial variability of PM2.5. To address this limitation, some researchers utilized satellite remote sensing data as inputs [7,14,16]. Chudnovsky et al. [7] utilized satellite data that is the high-resolution (1 km) aerosol optical depth (AOD) retrieval from the moderate resolution imaging spectroradiometer (MODIS) data. The study used the day-specific calibrations of AOD data for predicting PM2.5. Furthermore, the study demonstrated that the accuracy of prediction of PM2.5 increases by adding sufficient meteorological and land-use data. Zamani et al. [16] utilized the ground measurements of PM2.5, the meteorological data, and the remote sensing AOD data as the inputs. They investigated the feature importance for predicting PM2.5 concentrations using the random forest, eXtreme Gradient Boosting (eXGB), and deep neural network approaches. Similarly, Shtein et al. [14] utilized the satellite remote sensing data to improve the prediction of PM2.5. They proposed an ensemble model to adopt the advantages of each model to demonstrate that the ensemble model outperforms the individual models.

Although most studies have focused on outdoor PM concentrations, many residents in the metropolis use public transportation, including the subway where indoor air quality affects the health of riders. Park et al. [13] focused on indoor air quality of subway systems in the metropolis. However, it is difficult to obtain indoor PM data because of the deployment of the measurement systems. Thus, they predicted the indoor PM concentration using inputs such as outdoor PM10, the number of subway trains in operation, and information on ventilation operation. ANN was used to predict the indoor PM10, and the model empirically demonstrated a high correlation between the predicted and the measured values. Furthermore, they investigated the relations between the performance of the ANN model and the depth of the underground subway station.

## 3. Prediction Model of Particulate Matter Concentration

This section introduces our modeling approach using Gaussian Process Regression (GPR). Before discussing the GPR, we briefly review our baseline model, ARIMA.

### 3.1. Prediction Models

#### Vector Autoregressive Integrated Moving Average with Linear Regression (Varima + Lr)

The ARIMA is a method used to predict continuous outputs with a time series dataset. ARIMAs are generalizations of autoregressive moving average (ARMA) models wherein the concept of *integration* is added. The ARMA model is a combination of auto-regression (AR) and moving average (MA) models. The ARMA model is denoted by ARMA(p,q), that is, the combination of AR(p) and MA(q). The autoregressive moving average model ARMA(p,q) with orders *p* and *q* is given by

(1) $$\begin{array}{c} y_{t}=c+\sum_{i=1}^{p}\alpha_{i}y_{t-i}+\sum_{j=1}^{q}\theta_{j}\epsilon_{t-j}+\epsilon_{t} \end{array}$$

where *c* is a constant; $\alpha_{1},...,\alpha_{p}$ are the regression coefficient parameters of the AR model; $\theta_{1},...,\theta_{q}$ are the weight parameters of the MA model; and $\epsilon_{t},\epsilon_{t-1},...,\epsilon_{t-q}$ are the error terms sampled from a normal distribution with zero μ and an arbitrarily chosen σ. Furthermore, $y_{t}$ is the observed PM concentration to be estimated.

To address the limitation from the non-stationarity, *Integration* (or Differencing) is applied to the ARMA model to enable the non-stationary time series data follow the stationary property called ARIMA. For example, the first differencing $y_{t}^{{}^{\prime }}$ of $y_{t}$ is computed as

(2) $$\begin{array}{c} y_{t}^{{}^{\prime }}=y_{t}-y_{t-1}. \end{array}$$

Denoting the *d*-th differencing of $y_{t}$ by $y_{t}^{\left(d\right)}$, the ARIMA model ARIMA(p,d,q), with orders p,d,q, is given by

(3) $$\begin{array}{c} y_{t}^{\left(d\right)}=c+\sum_{i=1}^{p}\alpha_{i}y_{t-i}^{\left(d\right)}+\sum_{j=1}^{q}\theta_{j}\epsilon_{t-j}+\epsilon_{t} \end{array}$$

where *d* is called the degree of differencing.

### 3.2. Prediction on Diverse Locations

There are several monitoring sites of PM in the metropolitan area. That is, there are multiple output values, $y_{t}$’s, measured by the different observatories at time *t*. To describe the spatial dependencies over the observations, the Vector ARIMA model (VARIMA) extends the ARIMA model [21]. Whereas $y_{t}$ in ARIMA represents the observation data at time *t* from a single source, VARIMA uses $y_{t}^{i}$ to represent the observation data, measured by the *i*-th monitoring site at time *t*. For simplicity, we denote the output value as $y_{t}^{i}$ regardless of the level of differencing in ARIMA. Then, the VARIMA model VARIMA(p,d,q) with orders p,q,d, is given by

(4) $$\begin{array}{c} y_{t}^{i}=c+\sum_{k=1}^{p}\sum_{j=1}^{S}\alpha_{i,j}^{k}y_{t-k}^{j}+\sum_{k=1}^{q}\sum_{j=1}^{S}\theta_{i,j}^{k}\epsilon_{t-k}^{j}+\epsilon_{t}^{i}\;for\;i=1,...,S, \end{array}$$

where *S* is the number of the monitoring sites; $\alpha_{i,j}^{k}$ are the extended regression coefficient parameters of the AR model that considers the spatial dependencies (*i* and *j*-th monitoring sites) over the observations under the degree of *k* over *p*; $\theta_{i,j}^{k}$ are the extended weight parameters of the MA model that considers the dependencies (*i* and *j*-th monitoring sites) over the error terms under the degree of *k* over *q*. Owing to the extension to the vector space, the VARIMA model considers the spatial dependencies over output values from different monitoring sites, while maintaining the advantages of the ARIMA model. From the aforementioned equations of VARIMA, we can also extend the AR,MA, and ARMA models to the vector space, denoted by VAR, VMA, and VARMA, respectively. It is noted that VARIMA(p,d,q) is a generalization of VAR(p), VMA(q), and VARMA(p,q).

Furthermore, we combine the linear regression model with VARIMA to incorporate the site-specific perspectives of input features, such as the topography, and the meteorological data. We denote this model by VARIMA + LR. The role of the parameters in the linear regression model is to investigate the relations between input features and the corresponding output values. We assume that the relations do not depend on the monitoring sites. Therefore, we utilize the same parameters with respect to the linear regression model’s total output dimensions that means the same parameters for the *N* monitoring sites. Therefore, the combined model, VARIMA(p,q,d)+LR, is given by

(5) $$\begin{array}{c} y_{t}^{i}=c+\sum_{k=1}^{p}\sum_{j=1}^{S}\alpha_{i,j}^{k}y_{t-k}^{j}+\sum_{k=1}^{q}\sum_{j=1}^{S}\theta_{i,j}^{k}\epsilon_{t-k}^{j}+\sum_{j=1}^{M}\phi_{j}x_{i,t}^{j}+\epsilon_{t}^{i} \end{array}$$

where *M* is the number of input features used. Herein, we denote the input feature by $x_{i,t}^{j}$ representing the *j*-th input feature information observed at the *i*-th monitoring site at time *t*. Furthermore, $\phi_{j}$ is the linear regression parameter corresponding to the input feature $x_{i,t}^{j}$ given *i* and *t*. In matrix notation, VARIMA(p,d,q)+LR is given by

(6) $$\begin{array}{c} Y_{t}=\left[\begin{array}{c} y_{t}^{1}\\ y_{t}^{2}\\ ...\\ y_{t}^{S} \end{array}\right]=\left[\begin{array}{cccc} \alpha_{1,1}^{1} & \alpha_{1,2}^{1} & ... & \alpha_{1,S}^{1}\\ \alpha_{2,1}^{1} & \alpha_{2,2}^{1} & ... & \alpha_{2,S}^{1}\\ ... & ... & ... & ...\\ \alpha_{S,1}^{1} & \alpha_{S,2}^{1} & ... & \alpha_{S,S}^{1} \end{array}\right]\left[\begin{array}{c} y_{t-1}^{1}\\ y_{t-1}^{2}\\ ...\\ y_{t-1}^{S} \end{array}\right]+...+\left[\begin{array}{cccc} \alpha_{1,1}^{p} & \alpha_{1,2}^{p} & ... & \alpha_{1,S}^{p}\\ \alpha_{2,1}^{p} & \alpha_{2,2}^{p} & ... & \alpha_{2,S}^{p}\\ ... & ... & ... & ...\\ \alpha_{S,1}^{p} & \alpha_{S,2}^{p} & ... & \alpha_{S,S}^{p} \end{array}\right]\left[\begin{array}{c} y_{t-p}^{1}\\ y_{t-p}^{2}\\ ...\\ y_{t-p}^{S} \end{array}\right]\\ \;+\left[\begin{array}{cccc} \theta_{1,1}^{1} & \theta_{1,2}^{1} & ... & \theta_{1,S}^{1}\\ \theta_{2,1}^{1} & \theta_{2,2}^{1} & ... & \theta_{2,S}^{1}\\ ... & ... & ... & ...\\ \theta_{S,1}^{1} & \theta_{S,2}^{1} & ... & \theta_{S,S}^{1} \end{array}\right]\left[\begin{array}{c} \epsilon_{t-1}^{1}\\ \epsilon_{t-1}^{2}\\ ...\\ \epsilon_{t-1}^{S} \end{array}\right]+...+\left[\begin{array}{cccc} \theta_{1,1}^{q} & \theta_{1,2}^{q} & ... & \theta_{1,S}^{q}\\ \theta_{2,1}^{q} & \theta_{2,2}^{q} & ... & \theta_{2,S}^{q}\\ ... & ... & ... & ...\\ \theta_{S,1}^{q} & \theta_{S,2}^{q} & ... & \theta_{S,S}^{q} \end{array}\right]\left[\begin{array}{c} \epsilon_{t-q}^{1}\\ \epsilon_{t-q}^{2}\\ ...\\ \epsilon_{t-q}^{S} \end{array}\right]\\ +\left[\begin{array}{cccc} x_{t,1}^{1} & x_{t,2}^{1} & ... & x_{t,M}^{1}\\ x_{t,1}^{2} & x_{t,2}^{2} & ... & x_{t,M}^{2}\\ ... & ... & ... & ...\\ x_{t,1}^{S} & x_{t,2}^{S} & ... & x_{t,M}^{S} \end{array}\right]\left[\begin{array}{c} \phi_{1}\\ \phi_{2}\\ ...\\ \phi_{M} \end{array}\right]+\left[\begin{array}{c} \epsilon_{t}^{1}\\ \epsilon_{t}^{2}\\ ...\\ \epsilon_{t}^{S} \end{array}\right]. \end{array}$$

#### Gaussian Process Regression

Formally, GPR uses a GP prior defined over functions p(f), where *f* is a function mapping from an input space $X\in R^{M}$ to R. Consider a set of arbitrary input points $X=\{x_{1},x_{2},...,x_{N}\}$ that can be past records of the PM concentration, X can be defined over space and time. Herein, we define X to be past records over the space and time, simultaneously; hence, the index of X has two axes corresponding to time and space, and use the notation $x_{n}\in R^{M}$ for $x_{i_{space},t_{time}}$ for simplicity. In addition, we write $y_{n}\in R$ for $y_{t_{time}}^{j_{space}}$ for simplicity.

After setting $X=\{x_{1},x_{2},...,x_{N}\}$, the corresponding set of random function variables is $f=\{f_{1},f_{2},...,f_{N}\}$. Given a pair of two input instances, the GP prior is defined by $f\left(x\right)∼\mathrm{GP}(m\left(x\right),K\left(x_{i},x_{j}\right))$ with the mean function, m(x); and the covariance function, $K(x_{i},x_{j})$ over the function f(x). The mean and covariance function are defined as follows:

(7) $$\begin{array}{cc} m(x) & =E[f(x)],\\ K(x_{i},x_{j}) & =E[\left(f\left(x_{i}\right)-m\left(x_{i}\right)\right)\left(f\left(x_{j}\right)-m\left(x_{j}\right)\right)]. \end{array}$$

A useful property of GP is the definition of the following joint multivariate Gaussian distribution, given any finite set of input points:

(8) $$\begin{array}{c} p(f|X)=N(f|m,K), \end{array}$$

where $m=(m\left(x_{1}\right),...,m\left(x_{N}\right))$ is the mean vector of input points; and the covariance function K is constructed from a covariance function $K(x_{i},x_{j})$. The covariance function, K, shows the domain information, such as proximity and temporal trends that are formulated as Matérn or squared exponential, or a customized function from the domain.

Using the prior function defined over the continuous domain on space and time, we introduce a GPR that plays a crucial role in estimating the PM concentration. Let $D=\left\{{\left(x_{i},y_{i}\right)}_{i=1}^{N}\right\}=\left(X,y\right)$ be a dataset consisting of snap-shot feature inputs X, such as windspeed, and the concentration of NOx; and the corresponding outputs y that is the concentration of the PM. To estimate the underlying function f:X→y, we assume $y_{i}=f\left(x_{i}\right)+\epsilon$, a noisy realization of the function from $f(x_{i})$, wherein $\epsilon ∼N(\epsilon |0,\sigma^{2})$ is the independent Gaussian noise.

In a typical regression scenario, given test points $x_{*}$, we estimate the corresponding function values $f_{*}$. Introducing a zero-mean GP prior over f(·) (Because the GP prior requires the zero-mean, the predicted values and the past records of the PM concentration should be normalized to have zero mean. Additionally, it is noted that the high variance in the GP prior will result in a numerical error in the GP sampling.) and using standard GP methodologies, we can derive the following predictive relationships to estimate $f_{*}$:

(9) $$\begin{array}{cc} p(y|f) & =N(y|f,\sigma^{2}I),\\ p(f_{*}|x^{*},X,y) & =N(f_{*}|\mu_{*},\Sigma_{*}), \end{array}$$

where the mean $\mu_{*}$ and covariance $K_{*}$ are defined as follows:

(10) $$\begin{array}{cc} \mu_{*} & =K_{*}K_{y}^{-1}y,\\ \Sigma_{*} & =K_{**}-K^{T}K_{y}^{-1}K_{*}. \end{array}$$

The covariance functions $K_{*}$, $K_{**}$, and $K_{y}$ are computed using the following formulae:

(11) $$\begin{array}{cc} K_{*} & =K(x,x_{*}),\\ K_{**} & =K(x_{*},x_{*}),\\ K_{y} & =K\left(x,x\right)+\sigma^{2}I. \end{array}$$

Before predicting the test points, we estimate the kernel hyperparameters by maximizing the marginal likelihood p(y|X)=∫p(y|f,X)p(f|X)df. Under the GPR model, the log-marginal likelihood is as follows:

(12) $$\begin{array}{c} \log p(y|X)=-\frac{1}{2}yK_{y}^{-1}y-\frac{1}{2}\log|K_{y}|-\frac{N}{2}\log2\pi . \end{array}$$

Because the maximization of the likelihood is a non-convex optimization task, we use standard gradient methods (The gradient can be computed using recent probabilistic programming frameworks, i.e., TensorFlow. In addition, one can use the matrix derivative to calculate $\frac{\partial }{\partial \theta }\log p(y|X)$ using *scikit-learn*). For arbitrary kernel hyperparameters θ, we obtain the partial derivatives of the log-marginal likelihood with respect to the hyperparameters:

(13) $$\begin{array}{c} \frac{\partial }{\partial \theta }\log p(y|X)=\frac{1}{2}y^{T}K_{y}^{-1}\frac{\partial K_{y}}{\partial \theta }K_{y}^{-1}y-\frac{1}{2}\mathrm{tr}\left(K_{y}^{-1}\frac{\partial K_{y}}{\partial \theta }\right). \end{array}$$

In the actual experiments, we required a further scaling for the GP implementation. Therefore, we used the Stochastic Variational Gaussian Process (SVGP) model [22] that scaled the model by inducing points from stochastic variational perspective. We initialized the inducing points from the training dataset using the K-Means Clustering algorithm by setting K to be 500.

To significantly approximate an arbitrary function, the GP should be designed with a kernel function, K, adapted to the problem domain. Our task of predicting the PM concentration is spatially clustered with strong temporal dependencies. This means that the PM concentration should be modeled with a joint distribution of temporal and spatial features. Moreover, there is a seasonal effect in the temporal pattern, and there are unaccounted outside effects, which will be treated as noise. Therefore, the periodicity is modeled in the kernel. Because there is no exact prior knowledge of the customized kernel on these settings, we composed a concatenated kernel function by varying our selection among Periodic, Matérn 3/2, Matérn 1/2, and RBF (Radial Basis Function) per feature variable. Whereas we enumerate the individual kernel function for each feature variable, the final composition of a kernel function is a weighted linear concatenation of these individual kernel functions mapped to the input features:

- Periodic kernel
  (14) $$\begin{array}{c} K_{Period}\left(x_{i},x_{j}\right)=\sigma^{2}\exp\left[-\frac{1}{2}\sum_{k=1}^{M}{\left(\frac{\sin\left(\frac{\pi }{p}\left(x_{i}^{k}-x_{j}^{k}\right)\right)}{\rho }\right)}^{2}\right], \end{array}$$
- RBF kernel
  (15) $$\begin{array}{c} K_{\mathrm{RBF}}\left(x_{i},x_{j}\right)=\exp\left(-\frac{d^{2}}{2\sigma^{2}}\right), \end{array}$$
- Matérn 1/2 (M12) kernel
  (16) $$\begin{array}{c} K_{M12}\left(x_{i},x_{j}\right)=\sigma^{2}\exp\left(-\frac{d}{\rho }\right), \end{array}$$
- Matérn 3/2 (M32) kernel
  (17) $$\begin{array}{c} K_{M32}\left(x_{i},x_{j}\right)=\sigma^{2}\left(1+\frac{\sqrt{3}d}{\rho }\right)\exp\left(-\frac{\sqrt{3}d}{\rho }\right), \end{array}$$

where $d=d\left(x_{i},x_{j}\right)=\sqrt{\sum_{k=1}^{M}{\left(x_{i}^{k}-x_{j}^{k}\right)}^{2}}$ is the distance metric between two data points; $\sigma^{2}$ is the output variance; *p* is the period; and ρ is the length scale.

To explain our kernel function design, we need to enumerate the modeled variables in our scenario. Table 2 shows the list of variables with annotations on their relevant types. Although most variables are frequently utilized features in studies [10,12,15,17,19,20], to our knowledge, there are no prior studies on developing a GPR model with topographic information, traffic information, ultraviolet information, and power plant operation information. Variables, such as wind direction and topographic categories, require further explanations because these two variables are converted into a set of dummy variables by the discretization. Wind direction is discretized in four directions resulting in four categorical variables of $X_{7},...,X_{10}$, and topographic categorization is a categorical variable resulting in dummy variables $X_{12},...,X_{15}$. The details of each variable will be discussed in Section 3.3. Table 3 presents details of our kernel function designs for the input features.

From examining the input variable list, we propose a kernel function for each input variable as shown in Table 3. Some variables provide complete information in pairs, i.e., Latitude($X_{1}$) and Longitude($X_{2}$); accordingly, such variables become a vector of kernel function inputs. We used the periodic kernel for the temporal inputs, and the other continuous inputs are processed by the Matèrn kernel function.

### 3.3. Input Data for the Prediction Model

Because of the limitation on data availability and our methodology, we limit our study to the Seoul metropolis and its surroundings. Seoul is approximately 600 km^2^ in size with a resident population of approximately 10 million. To estimate the concentration of the PM, and to investigate the relation between PM and other factors, we selected the various input features that were collected from different sources. The following subsections describe the input features in detail.

**Figure 1:**
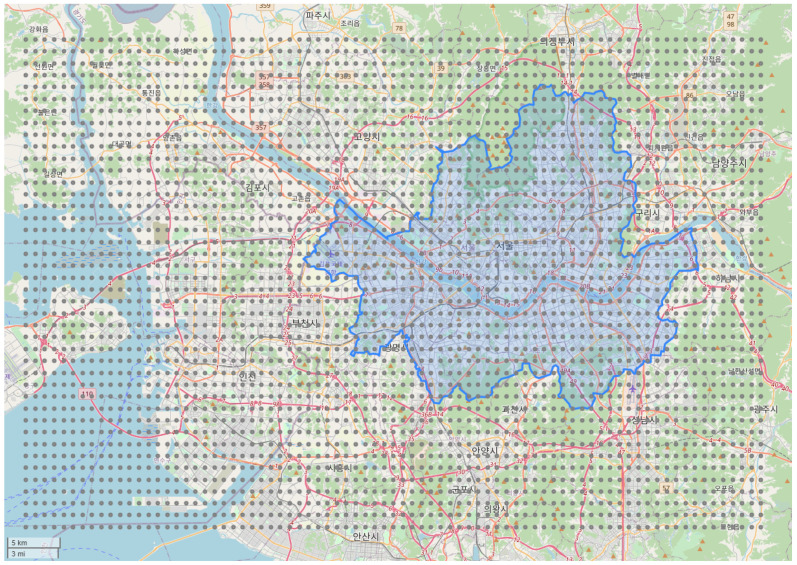
Grid setting of research area where the blue area represent Seoul, the grey dot indicates the position of each grid point, and the red lines are the major highways in the area.

To manage the data efficiently, we partitioned the study area into the grid cells with 0.01 degree latitude and longitude. Figure 1 represents our grid setting over the study area. The study area consists of total of 3318 cells, including the Seoul area.

The objective of this study is to predict the PM2.5 and PM10 concentrations of each grid cell using the suggested input features. Because we utilized GPR as our methodology, our study differs from other studies by the selection and diversity of the input features. Therefore, we enumerate each feature in the following subsections to describe the detailed information of each input data. In addition, we note that we excluded the modeling on industry types, such as chemical and metallurgy industries because the given region does not host such industries with significance.

#### 3.3.1. Particulate Matter and Air Quality Data

**Figure 2:**
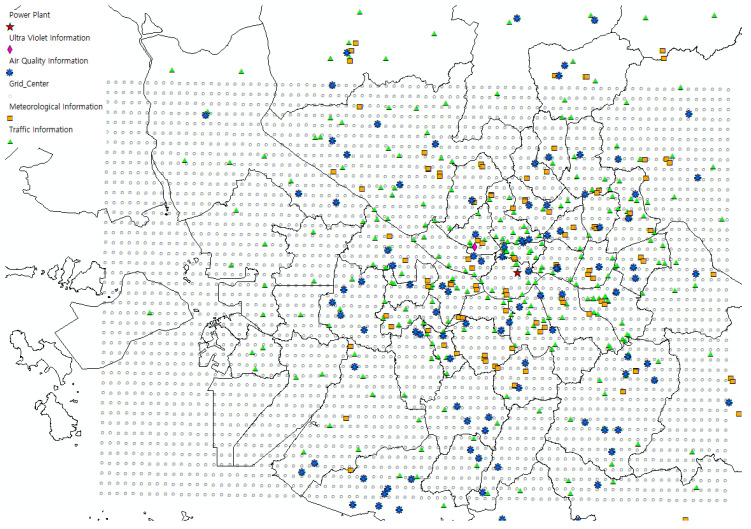
Locations of monitoring sites of each input type.

We utilized the PM and air quality data from the Korea Environment Corporation (https://www.airkorea.or.kr) that contain hourly data on several air quality elements including PM2.5 (μg/m^3^) yearly. In accordance with the regulations of the Korea Environment Corporation, only data up to the end of 2018 was available. Thus, we utilized the data of 2017 and 2018 for this study. There are 131 monitoring sites in our study area, and each site has several types of air sensors. Figure 2 shows the air quality monitoring sites as blue points. Owing to the breakdown and lack of sensors, the data have significantly high missing values, see Table 4. Therefore, we interpolated these missing values by averaging the values from other centers within 10 km, which is the optimal distance with the least interpolation errors among the five distance levels that we experimented. After interpolation, the missing value proportions of PM2.5 were reduced from 40.15% to 1.61% for the year 2017, and from 11.88% to 0.04% for the year 2018. From the air quality data, the hourly values of PM10 and PM2.5 were used as the target outputs measure. However, CO, NO_2_, O_3_, and SO_2_ values were used as the input variables of our forecasting model to consider the air quality condition.

#### 3.3.2. Location and Time

Additionally to the air quality data, we select the first two types of input variables as location and time. The location information includes the latitude and longitude of each grid cell for the analyzed area. The time information contains (1) the day of the year and (2) the hour of the day when the data was observed.

#### 3.3.3. Meteorological Data

We utilized the meteorological data that were measured hourly by an automatic weather system (AWS) from Korea Meteorological Administration (http://data.kma.go.kr). They provided the nine types of the meteorological information, namely (1) temperature, (2) wind speed, (3) wind direction, (4) precipitation, (5) spot-atmospheric pressure, (6) sea-level pressure, (7) humidity, (8) the ultraviolet strength from the sun, and (9) the illumination strength from the sun. However, we considered temperature, wind speed, wind direction, and precipitation as the input features regarding selecting variables for the meteorological data. Moreover, the other variables have many missing values in the observation data, which are not suitable for use as input variables. The main challenge is the mismatch between the locations of the observatories and the PM monitoring sites. To address this difference, we assigned the weather condition of the nearest observatory for each PM monitoring site.

#### 3.3.4. Topographic Data

**Figure 3:**
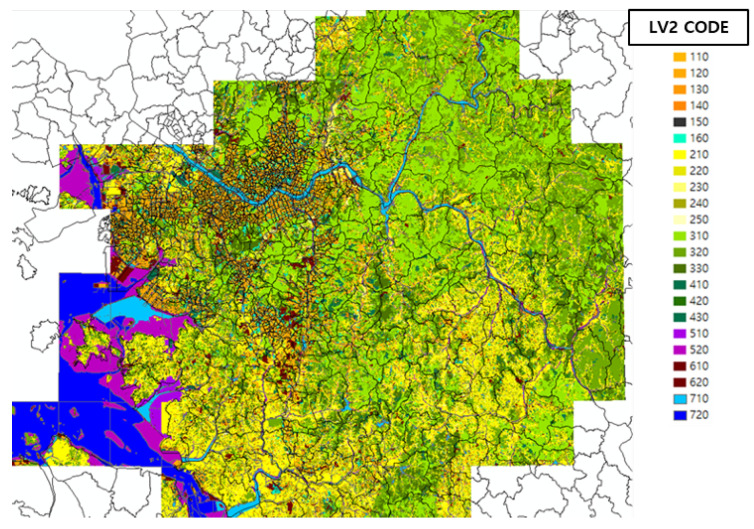
Land cover map of Seoul.

The Ministry of Environment (ME) provides the levels of the topographic information from the Environmental Spatial Information Service (https://egis.me.go.kr/bbs/landcover.do). In our setting, we utilized the level 2 code among the levels provided. To manage the level efficiently, we reduced to the five types of topographic categories, namely, urban, grassland, forest, water, and unknown areas. We introduced four dummy variables to represent the five categories. This means that each category has a binary indicator at the corresponding dimension except that the unknown area has all zero values. Thereafter, the topographic information is assigned to the corresponding grid cell as an input feature. Figure 3 shows the land cover map representing the topographic information of the level 2 code over our grid setting. Table 5 presents the topographic categories based on the level 2 code from the Environmental Spatial Information Service and its corresponding dummy variables. The use of dummy variables is common in structuring a regression model with categorical inputs [23,24,25].

#### 3.3.5. Traffic Data

Considered as a major metropolis in Korea, our study area has a complex road network. The traffic data are collected from the traffic points and junctions of major highways and roads, and a total of 406 traffic sensors provide hourly traffic data. Although this number is large, the sensors are sparsely located compared to the entire study area. We interpolated the traffic data from 406 traffic observation posts into grid level traffic information. To achieve a smooth interpolation of the traffic effect, we conducted a simple GPR for the traffic data using only spatio-temporal variables such as the latitude, and longitude; and the hour of the day.

#### 3.3.6. Ultraviolet Information

To investigate the relation between PM and the chemical reactions, we utilized the UV values as the input features. UV is partitioned into UVA (315–400 nm), UVB (280–315 nm), and UVC (100–280 nm) based on the wavelength. The UVA and UVB affect the surface of the earth, therefore, we collected the UVA and UVB data from the Korea Meteorological Administration (http://data.kma.go.kr). They provided the total quantity and the maximum quantity of UVA and UVB measured hourly. The area unit of the observed UV data is quite large such as Seoul. The area of Seoul and Anmyeon-do that they provide as unit area covers our grid cells entirely. Thus, the UV data from Seoul and Anmyeon-do were used for the UV variables, i.e., all grids in Seoul utilize the same UV data.

#### 3.3.7. Power Plant Data

As power plant data, we utilized the thermal power plant data that were collected from the Korea Power Exchange (https://kpx.co.kr). They only provided the information of the thermal power generation and the raw materials such as coal, gas, and oil (https://www.komipo.co.kr/kor/content/39/main.do?mnCd=FN021302), without the distinctions of plant type and built year for the entire Seoul area that is measured hourly. This measurement is also applied to the entire list in our grid cells. The other power plant data except for the thermal plant data was not available because they do not provide hourly measured data or do not open data to the public.

### 3.4. Performance Indicator of the Forecasting Model

Given the prediction methodology and the input variable list, we adopted two performance measurements that are frequently utilized in the domain.

#### 3.4.1. Root Mean Squared Error (RMSE)

Given that y is the actual observed value and $\hat{y}$ is the estimated value of the forecasting model, the mean squared error (MSE) measures the average of the squared errors. Herein, the errors are the average of the squared differences between the estimated values and the actual value. By taking the square root of MSE, the root mean squared error is computed as follows:

(18) $$\begin{array}{c} RMSE\left(y,\hat{y}\right)=\sqrt{\frac{1}{N}\sum_{i=1}^{N}{\left(y_{i}-\hat{y_{i}}\right)}^{2}} \end{array}$$

where *N* is the number of instances. The smaller value of RMSE indicates the higher predictive power of the model.

#### 3.4.2. Index-of-Agreement (IOA)

IOA represents the degree of the prediction error of the prediction model, varying between 0 and 1. IOA measures the ratio of the total mean square error, $\sum_{i=1}^{N}{\left(y_{i}-\hat{y_{i}}\right)}^{2}$, and the total potential error, $\sum_{i=1}^{N}{\left(y_{i}-\bar{y}\right)}^{2}+\sum_{i=1}^{N}{\left({\hat{y}}_{i}-\bar{y}\right)}^{2}$. By subtracting the ratio value from 1, IOA is computed as follows:

(19) $$\begin{array}{c} IOA\left(y,\hat{y}\right)=1-\frac{\sum_{i=1}^{N}{\left(y_{i}-\hat{y_{i}}\right)}^{2}}{\sum_{i=1}^{N}{\left(y_{i}-\bar{y}\right)}^{2}+\sum_{i=1}^{N}{\left({\hat{y}}_{i}-\bar{y}\right)}^{2}} \end{array}$$

where $\bar{y}$ is the average of the observation values. High IOA value indicates that the predicted values are consistent with the observed values.

## 4. Experiments

This section discusses the experimental components of our prediction model to predict the PM in the metropolis with a Gaussian Process.

### 4.1. Experimental Setting

**Figure 4:**
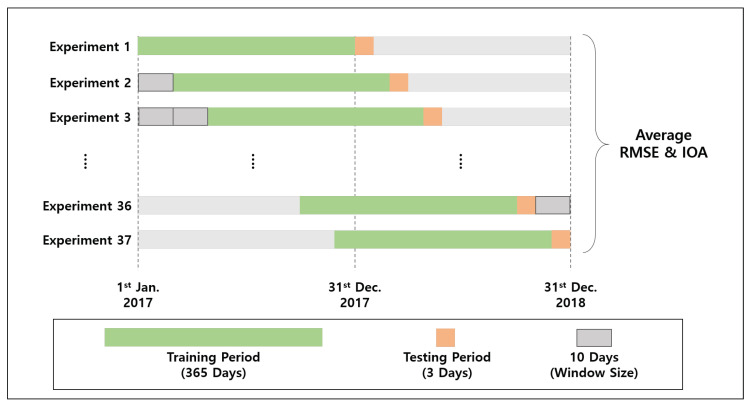
The experimental settings based on the sliding window approach where the window size is 10 days with 365 days of thetraining period and three days of the testing period.

Our goal is to predict PM2.5 and PM10 over our geospatial grids by training a GPR with observations from the monitoring sites. We utilized the input data that were observed in 2017 and 2018. Because the evaluation considers the temporal movement, we cannot simply perform N-fold cross validation. Therefore, we adopt the sliding window approach, described in Figure 4. We train the GPR with the 12-month observations from all monitoring sites. Thereafter, we test the GPR with the observations for the next three days. Subsequently, we move this 12-month training window toward the observation end time, which is the end of 2018. This moving window approach results in 37 replications because the window size is 10. In terms of the kernel designs of our GPR model, we combined the three kinds of kernels as presented in Table 3.

To examine the effectiveness of our GPR model, we implement three alternative statistical models, namely Linear Regression (LR) model, VARIMA model, and a combined model of LR and VARIMA (VARIMA+LR). Linear regression is a basic model to investigate the effect of each input. Because we considered a holistic perspective of the PM generation with respect to several input features, LR is suitable for a comparative investigation. A VARIMA model predicts the current PM concentrations from the previous. Because our model does not utilize the previous PM concentrations as an input, we implement VAIRMA+LR for unbiased comparison. Consequently, the VARIMA model admits the input features that we utilized. For variants of the VARIMA model, we implement the VAR, VMA, and VARMA models. We performed our experiments by varying the period of training sets and the types of output, such as PM2.5 and PM10.

### 4.2. Experimental Results

#### 4.2.1. Quantitative Results

Table 6 presents the performance of the models in terms of RMSE and IOA. RMSE is the error measurement. Therefore, lower values of RMSE are preferable. In contrast, IOA is the accuracy measurement, hence higher values of IOA are desired. From the experiments, GPR is preferable for both criteria of PM10 and PM2.5. Although the kernel choice causes a performance change, the change from the kernels is less than the difference between that from GPR and the variants of VARIMA. Comparing the variants of VARIMA, the VARIMA+LR model outperforms the VARIMA model in PM10. This means PM10 is significantly influenced by site-specific features, such as topography and meteorology. In spite of the better performance of VARIMA+LR in PM10, VARIMA+LR in PM2.5 is worse than VARIMA. Although, from literature, PM2.5 is affected by the local surroundings, our statistical analyses does not indicate this information. Therefore, features that contributed to predicting PM10 will be disjoint to the features for predicting PM2.5. This means that the features of PM2.5 should be investigated for further studies. We also note a consistent performance of LR and a weak performance of VARIMAs. We conjecture that a typical bias-variance trade-off is applicable to interpret the results. If a complex model is not well trained with a provided data, the prediction of the complex model becomes worse than its simpler models because of its high variance error with small bias improvement. From this perspective, the parameter inference of GPR showed a better training result given its highest performance, although GPR is the complex model in the compared model set.

Although the average RMSE of GPR is the lowest among the compared models, the relative significance of the error level should be compared. For this purpose, we also analyzed the IOA index that accounts for the scale of the target value. Our IOA is beyond 60% in the next three-day predictions, and recent studies focused on the same day or the next day forecasting [26,27].

#### 4.2.2. Temporal Patterns from Gaussian Process Regression

**Figure 5:**
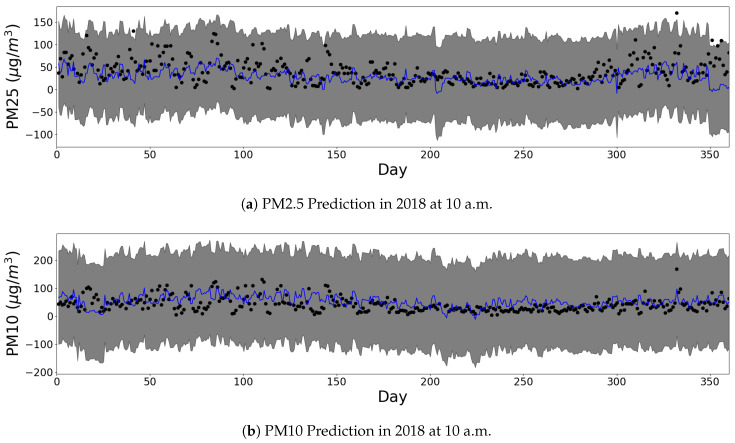
One year prediction results of our GPR model at grid location of (126.835169, 37.544682). (Black dot: observed, Blue line: predicted, Grey area: 95% confidence interval.)

**Figure 6:**
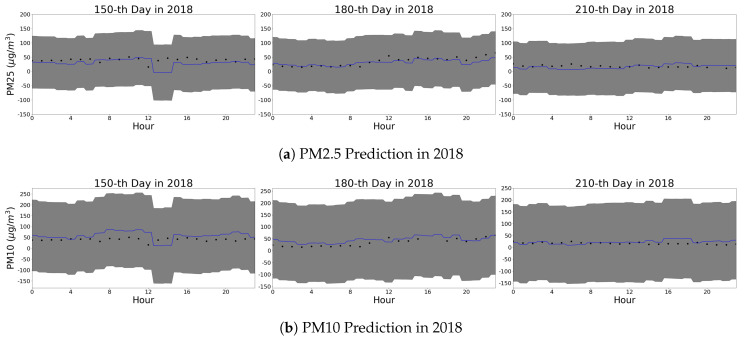
One day prediction results of our GPR model at grid location of (127.040207, 37.543796). (Black dot: observed, Blue line: predicted, Grey area: 95% confidence interval.)

Figure 5 and Figure 6 present the predictive abilities of GPR in some grid cell at several times. The black dots in figures indicate the observed points of PM2.5, and the blue line represents the predicted mean values of the GPR model. The grey area represents the 95% confidence interval at each time. Except for four days, the observations fall in the confidence interval throughout the year. From Figure 5, there is an insignificant seasonal trend that shows the up-turn on days 0–100 and days 300–364; and the down-turn on days 100–300. This corresponds to the winter and the summer seasons of the site, respectively.

Figure 6 shows three daily trends at an arbitrarily chosen site for PM10 and PM2.5, respectively. The intra-day trend is consistent with the lower PM concentration around 4:00 a.m.; and the higher PM concentration around 6:00 p.m. These correspond to the lowest activity within cities and the busiest traffic hour of the city.

We also examine the limitation of GPR that originates from the nature of the Gaussian distribution. The Gaussian distribution possesses a long tail from negative infinity to positive infinity. Therefore, the GPR does not predict if the input is always positive, or not. Figure 5 and Figure 6 show some areas of confidence interval in the negative PM2.5, which is unrealistic. In the experiments, the mean function of GPR is consistently positive. The high variance of the observations yields the wide confidence interval. We can minimize the confidence interval to be in the positive area of PM2.5 by adding sufficient observations in the deployment stage.

#### 4.2.3. Spatial Patterns from Gaussian Process Regression

**Figure 7:**
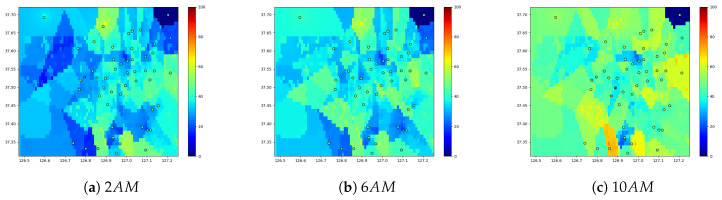
PM2.5 prediction at 45th day in 2018 over our grid.

Figure 7 shows the spatial patterns over Seoul. The dots indicate the observed PM2.5 concentration, and the other areas are covered with the predicted values from our GPR model. Figure 7 shows the prediction results at 2:00 a.m., 6:00 a.m., and 10:00 a.m. From 2:00 a.m. to 10:00 a.m., the overall PM2.5 concentration increases because the activities and traffic increases. The upper right subregion is the forest area, hence the PM2.5 concentrations in this area are consistently low. We observe that the commercial areas with several road segments have higher PM2.5 concentrations, whereas the mountain and the sea areas have low concentrations that shows the consistency between the prediction results and the topography of the city.

Figure 7 shows less variability in the observations than the predictions. The plotted observations originate from a specific timepoint of the modeled region, whereas the prediction is from the trained model of the entire past records. Therefore, the prediction shows the higher variability induced by the past records. This effect is illustrated in the upper right corner of the analyzed region. Although the corner is a forest area that has low PM concentrations over the period, the observation at a specific timestep can deviate from the historic pattern. Moreover, it be noted that, although the observation posts are clustered in the urban center that shows high variations in the predication, there are few observation posts in the suburban areas that have discrete changes by their topographies.

#### 4.2.4. Ablation Study

**Figure 8:**
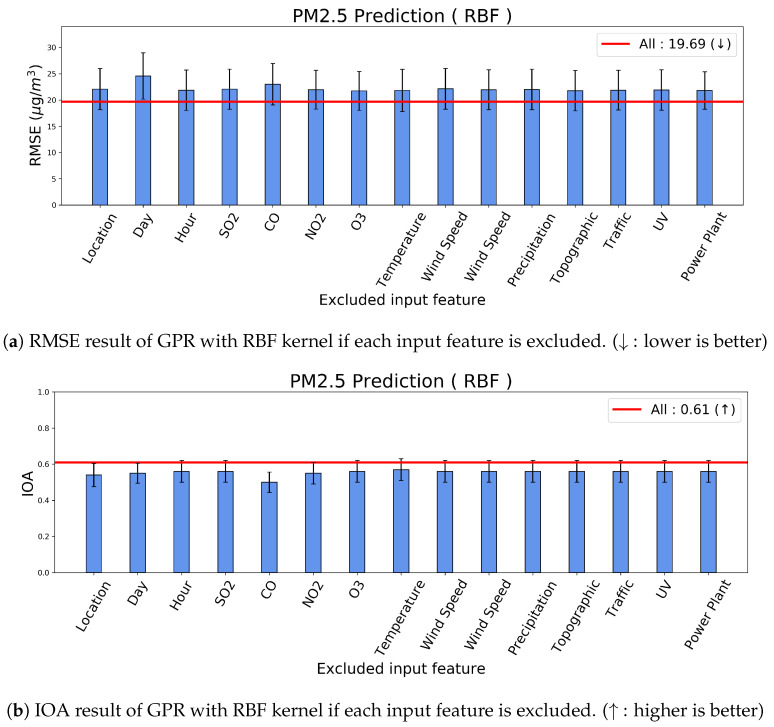
Ablation study for the input features in the prediction of PM2.5.

To investigate the relative importance of the input features, we conducted an ablation study based on our GPR model. From Table 3, we composed our kernel design by input features; thus, we implemented the ablated GPR models by excluding each kernel component corresponding to each input feature. Figure 8 presents the results of our GPR model for PM2.5 prediction; RMSE for Figure 8a; and IOA for Figure 8b. Each bar represents the prediction performance with a 95% confidence interval if the corresponding input feature was excluded. Therefore, the worse prediction performance indicates the value of the ablated input feature. Additionally, the red line represents the original performance of our model with all input features. All ablated cases reported worse performances than the original performance that indicates that the input features are necessary to estimate the concentration of PM2.5.

Particularly, the performance reduced significantly provided location ($X_{1},X_{2}$), say ($X_{3}$), and CO ($X_{20}$) are excluded. Therefore, these input features are relatively important for predicting PM2.5. Location is important because it determines the closeness to the traffic and the activities. Moreover, Time also becomes the indicator of the traffic and the activities, hence they latently infer the same dynamics. Another input from Time is seasonal effects, such as summer, and winter. Furthermore, CO is a highly correlated indicator of PM generation [28], hence CO is a key factor.

## 5. Conclusions

This study analyzes the capability of Gaussian process regression to predict the concentration of particulate matter. We recorded beyond 0.6 of IOA in the prediction of the next three days. GPR is versatile in including the input features with a customized kernel design. Furthermore, the GPR outperformed the VARIMA in the given prediction tasks. For example, the cyclic pattern of the seasonal trend can be captured by the periodic kernel. In addition, the spatial pattern is captured by the radial basis kernel function. In addition to the prediction performance, we identified the relatively important input features as Location, Time, and CO. We performed ablation studies to identify key features, and all features were necessary to statistically improve the IOA performances. Our study shows key feature selections from varying attributes in the prediction tasks. Therefore, analyses on the features is relevant information to actual modeling for the public system.

## Figures and Tables

**Table 1:** Summary of the existing forecasting models of the concentration of particulate matter with respect to complexity, methodology, independent variables, and dependent variable.

Previous Research			Independent Variables														Dependent Variables
Complexity	Research	Methodology	Location	Time	CO	NO_2_	O_3_	SO_2_	Temp- erature	Rain- fall	Wind Direction	Wind Speed	Topo- graphic	Traffic Volume	Ultra Violet	Power Plant	PM
Linear Model	Chudnovsky et. al [7]	AOD Retrieval + Regression	🗸	🗸						🗸		🗸					PM2.5
	Garcia et al. [8]	Generalized Linear Model	🗸	🗸	🗸	🗸	🗸	🗸	🗸	🗸	🗸	🗸					PM10
	Zhang et al. [9]	Spatio-temporal Land-use Regression	🗸	🗸					🗸	🗸			🗸				PM2.5
Neural Network Model	Lal et al. [10]	Vanilla ANN	🗸	🗸					🗸	🗸	🗸	🗸	🗸				PM10 PM2.5
	Lu et al. [11]	ANN + CPSO Algorithm	🗸	🗸					🗸	🗸		🗸		🗸			PM10 PM1
	Zhou et al. [12]	Recurren Fuzzy NN	🗸	🗸	🗸	🗸	🗸	🗸	🗸	🗸	🗸	🗸					PM2.5
	Park et al. [13]	Vanilla ANN	🗸	🗸													PM10
	Shtein et al. [14]	Ensemble model	🗸	🗸													PM10 PM2.5
	Zhao et al. [15]	LSTM-FC	🗸	🗸	🗸	🗸	🗸	🗸	🗸	🗸	🗸	🗸					PM2.5
	Zamani et al. [16]	Random Forest + eXGB + Deep NN	🗸	🗸					🗸	🗸		🗸					PM2.5
	Pak et al. [17]	CNN-LSTM	🗸	🗸	🗸	🗸	🗸	🗸	🗸	🗸	🗸	🗸					PM2.5
Nonlinear and Nonparametric Regression Model	Cheng et al. (2014) [18]	Gaussian Process Regression	🗸	🗸													PM2.5
	Reggente et al. [19]	Gaussian Process Regression	🗸	🗸	🗸	🗸	🗸	🗸									PM0.1
	Liu et al. [20]	Gaussian Process Regression	🗸	🗸	🗸	🗸			🗸								PM2.5
Nonlinear and Nonparametric Regression Model	Ours	Gaussian Process Regression	🗸	🗸	🗸	🗸	🗸	🗸	🗸	🗸	🗸	🗸	🗸	🗸	🗸	🗸	PM10 PM2.5

**Table 2:** Variable information.

Type	Variable	Information	Unit
Location	$X_{1}$	Latitude	Degree
	$X_{2}$	Longitude	Degree
Time	$X_{3}$	Day of Year	Year/Month/Day
	$X_{4}$	Hour of Day	h
Meteorological Information	$X_{5}$	Temperature	°C
	$X_{6}$	Precipitation	mm/h
	$X_{7}-X_{10}$	Wind Direction	Categorical
	$X_{11}$	Wind Speed	m/s
Topographic Information	$X_{12}-X_{15}$	Topographic Categories	Categorical
Traffic Information	$X_{16}$	Agent Traffic Volume	Vehicles/Hr
Air Quality Information	$X_{17}$	Sulfur Dioxide (SO_2_)	ppm
	$X_{18}$	Carbon Monoxide (CO)	ppm
	$X_{19}$	Nitrogen Dioxide (NO_2_)	ppm
	$X_{20}$	Ozone (O_3_)	ppm
Ultraviolet Information	$X_{21}$	UVA Max	MJ/m^2^
	$X_{22}$	UVA Sum	MJ/m^2^
	$X_{23}$	UVB Max	KJ/m^2^
	$X_{24}$	UVB Sum	KJ/m^2^
Power Plant	$X_{25}$	Usage of Thermal Power Plant	%

**Table 3:** Kernel design with respect to input variables.

Kernel	Information	Variables	Kernel Type 1 (Matérn)	Kernel Type 2 (RBF)	Kernel Type 3 (Matérn + RBF)
$k_{1}$	Latitude, Longitude	$X_{1},X_{2}$	Matérn 3/2 (M32)	RBF	RBF
$k_{2}$	Day of Year	$X_{3}$	Periodic	Periodic	Periodic
$k_{3}$	Hour of Day	$X_{4}$	Periodic	Periodic	Periodic
$k_{4}$	Temperature	$X_{9}$	Matérn 3/2 (M32)	RBF	RBF
$k_{5}$	Precipitation	$X_{10}$	Matérn 3/2 (M32)	RBF	Matérn 3/2 (M32)
$k_{6}$	Wind Direction	$X_{11},X_{12},X_{13},X_{14}$	Matérn 3/2 (M32)	RBF	RBF
$k_{7}$	Wind Speed	$X_{15}$	Matérn 3/2 (M32)	RBF	RBF
$k_{8}$	Topographic Categories	$X_{16},X_{17},X_{18},X_{19}$	Matérn 3/2 (M32)	RBF	RBF
$k_{9}$	Agent Traffic Volume	$X_{20}$	Matérn 3/2 (M32)	RBF	RBF
$k_{10}$	Sulfur Dioxide ($SO_{2}$)	$X_{5}$	Matérn 3/2 (M32)	RBF	Matérn 3/2 (M32)
$k_{11}$	Carbon Monoxide (CO)	$X_{6}$	Matérn 3/2 (M32)	RBF	Matérn 3/2 (M32)
$k_{12}$	Nitrogen Dioxide ($NO_{2}$)	$X_{7}$	Matérn 3/2 (M32)	RBF	Matérn 3/2 (M32)
$k_{13}$	Ozone ($O_{3}$)	$X_{8}$	Matérn 3/2 (M32)	RBF	Matérn 3/2 (M32)
$k_{14}$	Ultraviolet	$X_{21},X_{22},X_{23},X_{24}$	Matérn 3/2 (M32)	RBF	RBF
$k_{15}$	Usage of Thermal Power Plant	$X_{25}$	Matérn 3/2 (M32)	RBF	RBF

**Table 4:** The number of missing instances in each air quality elements.

	The Number of Missing Instances in Each Air Quality Data (%)						Total Instance
Year	${\mathrm{SO}}_{2}$	CO	$O_{3}$	${\mathrm{NO}}_{2}$	PM10	PM2.5	
2018	46,079 (4.16%)	55,436 (5.00%)	71,456 (6.44%)	50,039 (4.51%)	68,782 (6.20%)	131,734 (11.88%)	1,108,992
2017	31,978 (3.00%)	42,871 (4.02%)	58,319 (5.46%)	39,153 (3.67%)	47,856 (4.48%)	428,544 (40.15%)	1,067,304

**Table 5:** Topographic information.

LV2 Code	LV2 Name	LVL Code	LVL Name	Dummy Variable
110	Residential Area	1	Urban Area	[1, 0, 0, 0]
120	Industrial Area			
130	Commercial Area			
140	Amusement Facility Area			
150	Traffic Area			
160	Public Facilities Area			
610	Mining Area			
620	Artificial Area			
210	Rice Paddy Area	2	Grassland Area	[0, 1, 0, 0]
220	Farming Area			
230	House Farming Area			
240	Orchard Area			
250	Other Farming Area			
410	Natural Grassland Area			
420	Golf Course Area			
430	Other Grassland Area			
310	Broad-leaf Forest Area	3	Forest Area	[0, 0, 1, 0]
320	Coniferous Forest Area			
330	Mixed Forest Area			
510	Inland Wetland Area	4	Water Area	[0, 0, 0, 1]
520	Coastal Wetland Area			
710	Fresh Water Area			
720	Sea Water Area			
999	Unknown Area	5	Unknown Area	[0, 0, 0, 0]

**Table 6:** Quantitative results of the models.

Model	Model Specification	PM10		PM2.5	
		RMSE (μg/m^3^)	IOA	RMSE (μg/m^3^)	IOA
Linear Regression (LR)	LR	22.19 ± 4.65 (14.43)	0.56 ± 0.05 (0.15)	22.04 ± 5.08 (15.78)	0.55 ± 0.05 (0.15)
VAR(p)	VAR(1)	26.17 ± 5.07 (15.73)	0.26 ± 0.03 (0.08)	29.30 ± 6.63 (20.57)	0.21 ± 0.05 (0.16)
	VAR(2)	26.03 ± 4.75 (14.73)	0.29 ± 0.03 (0.08)	28.41 ± 6.29 (19.53)	0.22 ± 0.05 (0.15)
	VAR(3)	26.44 ± 4.36 (13.54)	0.29 ± 0.03 (0.09)	28.09 ± 5.85 (18.14)	0.23 ± 0.05 (0.14)
VMA(q)	VMA(1)	25.80 ± 5.01 (15.56)	0.26 ± 0.03 (0.09)	27.59 ± 5.78 (17.94)	0.21 ± 0.04 (0.12)
	VMA(2)	25.92 ± 5.00 (15.53)	0.27 ± 0.03 (0.09)	27.65 ± 5.75 (17.84)	0.22 ± 0.04 (0.12)
	VMA(3)	26.06 ± 4.99 (15.49)	0.28 ± 0.03 (0.09)	27.71 ± 5.72 (17.75)	0.23 ± 0.04 (0.12)
VARMA(p,q)	VARMA(1,1)	33.40 ± 4.46 (13.84)	0.30 ± 0.04 (0.13)	29.05 ± 6.15 (19.1)	0.30 ± 0.06 (0.18)
	VARMA(2,2)	45.78 ± 6.95 (21.57)	0.33 ± 0.04 (0.13)	28.11 ± 5.83 (18.1)	0.30 ± 0.05 (0.17)
	VARMA(3,3)	51.92 ± 7.45 (23.13)	0.35 ± 0.04 (0.11)	32.09 ± 6.56 (20.35)	0.32 ± 0.05 (0.15)
VARIMA(p,d,q)	VARIMA(1,1,1)	45.65 ± 7.01 (21.77)	0.15 ± 0.02 (0.05)	46.08 ± 7.02 (21.8)	0.10 ± 0.01 (0.04)
	VARIMA(2,1,2)	45.55 ± 7.01 (21.74)	0.20 ± 0.02 (0.06)	45.73 ± 6.99 (21.68)	0.14 ± 0.02 (0.06)
	VARIMA(3,1,3)	45.89 ± 6.98 (21.67)	0.22 ± 0.02 (0.06)	45.55 ± 6.95 (21.58)	0.16 ± 0.02 (0.06)
VAR(p)+LR	VAR(1)+LR	23.89 ± 4.55 (14.12)	0.53 ± 0.05 (0.17)	29.60 ± 4.75 (14.73)	0.41 ± 0.05 (0.15)
	VAR(2)+LR	23.56 ± 4.96 (15.4)	0.56 ± 0.05 (0.16)	31.11 ± 5.40 (16.75)	0.42 ± 0.05 (0.15)
VMA(q)+LR	VMA(1)+LR	21.04 ± 4.66 (14.47)	0.59 ± 0.05 (0.15)	25.60 ± 4.97 (15.41)	0.49 ± 0.05 (0.14)
	VMA(2)+LR	21.12 ± 4.65 (14.44)	0.59 ± 0.05 (0.15)	25.56 ± 4.96 (15.38)	0.49 ± 0.05 (0.14)
	VMA(3)+LR	21.20 ± 4.65 (14.42)	0.59 ± 0.05 (0.15)	25.52 ± 4.95 (15.36)	0.49 ± 0.05 (0.14)
VARMA(p,q)+LR	VARMA(1,1)+LR	47.79 ± 10.26 (31.84)	0.43 ± 0.05 (0.17)	46.62 ± 6.19 (19.22)	0.31 ± 0.04 (0.13)
	VARMA(2,2)+LR	53.82 ± 14.01 (43.47)	0.46 ± 0.04 (0.13)	54.28 ± 7.86 (24.38)	0.33 ± 0.05 (0.15)
	VARMA(3,3)+LR	60.49 ± 13.31 (41.3)	0.45 ± 0.04 (0.12)	55.35 ± 14.15 (43.9)	0.35 ± 0.05 (0.14)
VARIMA(p,d,q)+LR	VARIMA(1,1,1)+LR	50.65 ± 7.26 (22.53)	0.19 ± 0.02 (0.06)	44.93 ± 6.54 (20.3)	0.16 ± 0.02 (0.05)
	VARIMA(2,1,2)+LR	46.55 ± 7.07 (21.94)	0.23 ± 0.02 (0.06)	43.54 ± 7.18 (22.27)	0.20 ± 0.03 (0.08)
	VARIMA(3,1,3)+LR	45.86 ± 7.44 (23.1)	0.25 ± 0.02 (0.07)	45.65 ± 6.89 (21.38)	0.19 ± 0.02 (0.06)
Gaussian Process Regression	GPR - (Matérn)	21.10 ± 4.29 (13.33)	**0.61 ± 0.05 (13.93)**	21.96 ± 4.97 (15.42)	0.58 ± 0.05 (0.15)
	GPR - (RBF)	21.13 ± 4.28 (13.29)	0.59 ± 0.05 (13.78)	**19.16 ± 4.71 (14.63)**	**0.61 ± 0.04 (0.14)**
	GPR - (Matérn + RBF)	**20.97 ± 4.40 (13.67)**	0.60 ± 0.05 (13.92)	21.92 ± 4.89 (15.16)	0.57 ± 0.05 (0.15)

## Abbreviations

The following abbreviations are used in this manuscript:

PM	Particulate Matter
CO	Carbon Monoxide
NO_2_	Nitrogen Dioxide
SO_2_	Sulfur Dioxide
O_3_	Ozone
UV	Ultraviolet
RMSE	Root Mean Squared Error
IOA	Index Of Agreement
GP	Gaussian Process
GPR	Gaussian Process Regression
SVGP	Stochastic Variational Gaussian Process
RBF	Radial Basis Function
LR	Linear Regression
AR	Auto-Regressive Model
MA	Moving Average Model
ARMA	Autoregressive Moving Average Model
ARIMA	Auto-Regressive Integrated Moving Average
VAR	Vector Auto-Regressive Model
VMA	Vector Moving Average Model
VARMA	Vector Auto-Regressive Moving Average Model
VARIMA	Vector Auto-Regressive Integrated Moving Average Model
GLM	Generalized Linear Model
NN	Neural Network
PLS	Partial Least Square
LSTM	Long Short-Term Memory
FC	Fully Connected
CNN	Convolutional Neural Network
MLP	Multi-Layer Perceptron
UFP	Ultra-Fine Particle
CPSO	Chaotic Particle Swarm Optimization
ANN	Artifial Neural Network
AOD	Aerosol Optical Depth
MODIS	Moderate Resolution Imaging Spectroradiometer
eXGB	eXtreme Gradient Boosting
ME	Ministry of Environment
MDPI	Multidisciplinary Digital Publishing Institute
